# Challenges in the Vaccination of HIV-Infected Individuals

**DOI:** 10.3390/vaccines14010040

**Published:** 2025-12-29

**Authors:** Marta Sisteré-Oró, Roberto Güerri-Fernandez, Montserrat Plana, Gennady Bocharov, Andreas Meyerhans

**Affiliations:** 1 Infection Biology Laboratory, Department of Medicine and Life Sciences (MELIS), Universitat Pompeu Fabra (UPF), 08003 Barcelona, Spain; 2 Infectious Diseases Unit, Hospital del Mar Research Institute, 08003 Barcelona, Spain; 3 CIBER Enfermedades Infecciosas (CIBERINFEC), Instituto de Salud Carlos III, 28029 Madrid, Spain; 4 Department of Medicine and Life Sciences (MELIS), Universitat Pompeu Fabra (UPF), 08003 Barcelona, Spain; 5 AIDS Research Group, Institut d’Investigacions Biomèdiques August Pi i Sunyer (IDIBAPS), Universitat de Barcelona, 08036 Barcelona, Spain; 6 Marchuk Institute of Numerical Mathematics, Russian Academy of Sciences, 119333 Moscow, Russia; 7 Institute for Computer Science and Mathematical Modelling, Sechenov First Moscow State Medical University, 119991 Moscow, Russia; 8 Institució Catalana de Recerca i Estudis Avançats (ICREA), 08010 Barcelona, Spain

**Keywords:** people living with HIV (PLWH), vaccination, highly active antiretroviral therapy (HAART), immunosuppression, functional cure

## Abstract

Vaccination of people living with an HIV infection (PLWH) remains a worldwide challenge. The reason is the heterogeneity of this group that comprises people with and without highly active retroviral treatment and varying degrees of immunosuppression. In this review, we (i) highlight the impact of antiviral treatment success on vaccination outcomes, (ii) describe the current vaccine recommendations for PLWH, and (iii) summarize immunotherapeutic strategies for improved HIV immune control.

## 1. Introduction

The AIDS pandemic caused by the human immunodeficiency virus Type 1 (HIV-1) and Type 2 (HIV-2) continues to be a global health threat. It was first recognized in 1981 [1] and has claimed approximately 44.1 million lives worldwide [2]. Currently, an estimated 39.9 million people are living with HIV infection (PLWH), of whom about 1.3 million were newly infected in 2023 alone [3]. HIV targets mainly CD4+ T cells and macrophages. This leads, if left untreated, to progressive CD4+ T-cell depletion and immunosuppression [4,5]. Depending on the HIV subtype, the pace of HIV disease progression varies. HIV-1 is typically associated with more rapid CD4+ T-cell loss and higher levels of viremia, whereas HIV-2 infection generally follows a slower course with lower viral loads [6,7,8,9,10]. The AIDS stage of an HIV infection is reached when the CD4+ T cell count drops below 200 cells/µL of blood [11,12]. At this stage, the risk of life-threatening complications is markedly increased [11,13,14,15]. They include the appearance of opportunistic infections (OIs) like *Pneumocystis jirovecii*, *Mycobacterium tuberculosis*, Herpesviridae, and Cytomegalovirus, as well as AIDS-defining malignancies such as Kaposi sarcoma, invasive cervical cancer, and aggressive B-cell non-Hodgkin lymphomas [16,17]. With the rise in highly active antiretroviral therapy (HAART), disease progression significantly changed (see below). Nonetheless, PLWH often remain immunologically compromised, exhibiting more severe outcomes of bacterial or viral infections [13,14]. To counteract this, vaccination has become a critical and indispensable component of comprehensive care for PLWH (see below).

## 2. The Impact of HAART on HIV Infection

**Figure 1:**
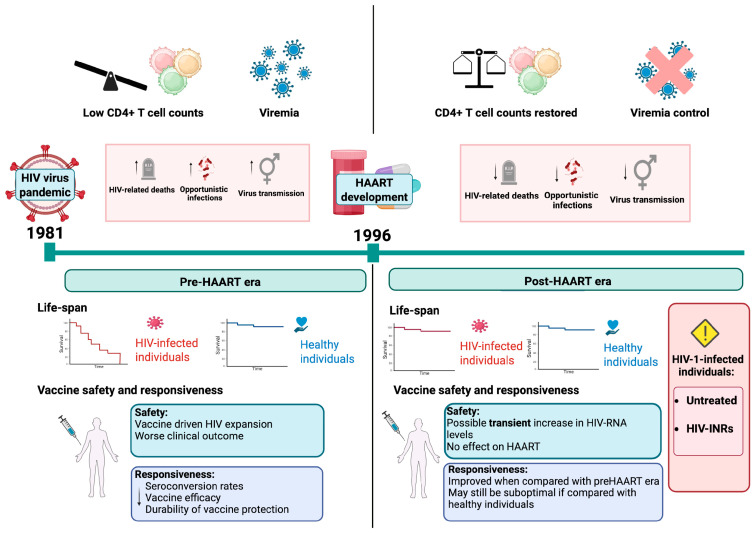
Antiviral-drug-mediated HIV control increased CD4+ T cell counts and enabled close to normal vaccination responses. The general characteristics of PLWH are given for the pre-HAART and post-HAART era. HAART enabled efficient viral suppression and restored CD4+T cell counts in the majority of HIV-infected individuals. This dramatically reduced HIV-related morbidity/mortality (achieving a near-normal lifespan) and facilitated close to normal vaccination responses. Nonetheless, special considerations are required for HIV-infected immunological non-responders and untreated individuals, who continue to show impaired immune functions. Abbreviations: HIV, human immunodeficiency virus; HAART, highly active antiretroviral therapy; HIV-INRs, HIV-infected immunological non-responders; PLWH, people living with HIV infection.

Antiviral drugs and protective vaccines are the two pillars against the spread of any viral infection. The development of the latter against HIV, despite over 40 years of intense research, has been so far unsuccessful, and there is no efficient HIV vaccine in sight [18,19,20]. Reasons are the extraordinary genetic diversity of HIV and its evasion of immune surveillance through immune escape and rapid integration into the host cell chromosome with establishment of latency [21,22,23,24]. In contrast, anti-HIV drug development is a success story. It started to boom in the mid-1990s with the production of HIV-1 protease inhibitors, which were combined with already-known nucleoside analogs and efficiently reduced virus loads in infected individuals [25,26]. The era of the so-called “Highly Active Antiretroviral Therapy” (HAART) was born, which enables the reduction in plasma HIV-1 RNA to undetectable levels (<50 copies/mL), albeit without eliminating latently infected virus reservoir cells [27,28,29]. HAART marked a paradigm shift in HIV management because it transformed the universally fatal HIV-induced disease into a manageable chronic condition [30,31] and allowed PLWH to participate in normal life [32,33,34] (Figure 1). Ever since, further anti-HIV drugs against different targets have been developed. Together, they currently sum up more than 30 individual antiviral agents and include reverse transcriptase inhibitors, protease inhibitors, integrase strand transfer inhibitors, entry inhibitors, and capsid inhibitors [35,36]. To simplify medication and improve adherence, pharmaceutical strategies have focused on developing combination drugs (over 20 are currently available) [37,38], and long-acting formulations [39,40]. An example of the latter is the injectable combination of Cabotegravir and Rilpivirine, which is administered every eight weeks [40]. Importantly, new drugs are in the pipeline and could be administered even less frequently, with targets for dosing every four and six months [41,42]. These drugs are essential as therapeutics in PLWH (controlling viral loads and reducing virus transmission) and pre-exposure prophylaxis (PrEP) in people at high risk of HIV infection [39,43,44,45]. All these advances in antiviral therapy are considered in the continuously updated treatment guidelines from the World Health Organization (WHO) [46,47]. Based on pivotal trials like START and TEMPRANO [48], they recommend early initiation of HAART regardless of the CD4+ T cell count. It improves outcomes of HIV infections by significantly reducing mortality, stopping progression to AIDS, and enabling immune recovery across diverse populations [48,49,50,51,52].

Importantly, not every HIV-infected individual benefits from the advances of HAART. As of 2023, about 23% of the 39.9 million PLWH worldwide—roughly 9.2 million individuals—remain untreated due to lack of drug access or lack of knowledge about their serostatus [2] (Figure 1). This treatment gap is particularly pronounced in certain regions and populations, perpetuating the clinical consequences of untreated HIV infection and complicating public health efforts [53]. Since HAART is the essential prerequisite for immune reconstitution (see below), the millions of untreated PLWH may lack the necessary CD4+ T cell numbers for mounting protective antibody and T-cell responses upon vaccination. This undermines the efficacy and public health benefit of vaccination campaigns in this cohort [5]. Furthermore, within the HAART-treated population itself, there are subgroups that, despite showing virologic control, remain in an immunosuppressed state and risk clinical sequelae [54]. Both groups may require special attention when it comes to protecting them from infectious diseases through vaccination (see below).

## 3. Challenges of Vaccinating HIV-Infected Individuals

Vaccination is the most efficient and cost-effective measure to protect an individual from pathogenic infections. To induce a protective vaccine response, the vaccinee needs to be immunocompetent and thus able to generate effective antibody and T-cell responses [55]. However, since HIV infection attacks CD4+ T cells, a key immune-response-coordinating cell type, it can significantly impair the immunocompetence of the host and affect vaccine responsiveness [5,56,57,58,59,60]. This may manifest as a lower immune response towards vaccination and a reduced duration of the response [61], and a constant skewing and restriction of specific T-cell immunity towards environmental antigens [62]. As all this is linked to HIV loads and CD4+ T cell counts, it remains to be determined at what levels, after HAART, the immunocompetence of PLWH can be considered as restored and possibly normal. A large epidemiological cohort study comparing all-cause mortality of over 80,000 treated HIV-1-infected adults (median age 37 years; 70% men) with the general population, adjusted for age and gender, provided important insights in this regard [33]. Excluding intravenous drug users, they found that the all-cause mortality of individuals with treated HIV infection and CD4+ T cell counts above 500 cells/µL of blood was similar to that of the general population [33]. In contrast, HIV-infected individuals with lower counts show distinct and increased infection susceptibilities [63]. Thus, individuals with CD4+ T cell counts above 500 cells/µL may be considered immunocompetent, comparable to uninfected controls. A similar cut-off of 500 CD4+ T cells/µL of blood has also been proposed for a new classification scheme of immunological responders versus non-responders after HAART [54]. According to this classification, immunocompetent responders (IR) are those PLWH who maintain >500 CD4+ T cells/µL of blood from baseline to after 24 months of therapy or who achieve >500 CD4+ T cells/µL of blood in that time frame. Second, patients who stay below 500 CD4+ T cells/µL but have a CD4+ T cell increase of >200 cells/µL are considered immunocompromised responders. Third, PLWH who stay below 500 CD4+ T cells/µL of blood and have a CD4+ T cell increase of less than 200 cells/µL of blood within 24 months of therapy are considered immunological non-responders (INR) [54]. The last two groups are immunocompromised, have an increased risk of opportunistic infections and neoplasms [64,65,66,67], and thus may require special attention when vaccinated [68,69,70,71,72,73,74,75] (Figure 1).

The proposed new classification scheme of PLWH after antiviral therapy is an important step towards harmonizing patient stratification [54]. However, it should be followed with an open eye for exceptions when evaluating immune responses after vaccinations. One should bear in mind that the risks of clinical progression or death of individuals who start HAART with CD4+ T cell counts below 200 cells/µL of blood remain increased even after reaching counts of 500 cells/µL [76]. Furthermore, an earlier study of the incidence of AIDS-defining illnesses concluded that immune reconstitution after HAART is not complete until CD4+ T cell counts increase to over 750 cells/µL of blood [77]. Accordingly, a close follow-up on PLWH is highly recommended.

## 4. Vaccine Recommendations for PLWH

**Figure 2:**
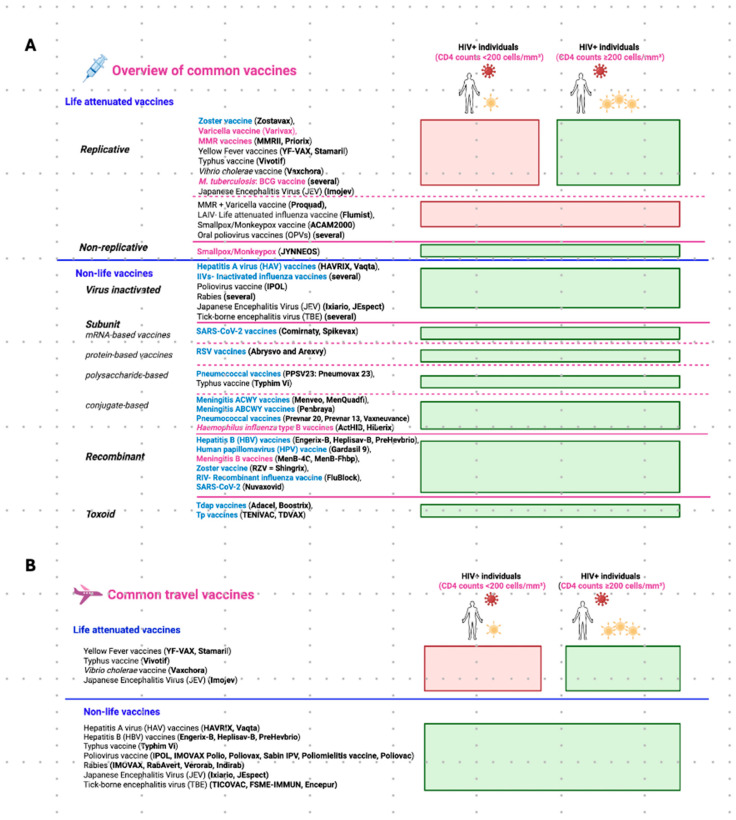
Common vaccines with safety profiles and recommendations for use in PLWH. Vaccines are listed according to their specific vaccine type. Their safe use in PLWH is indicated as green bars on the right. Contraindicated vaccines are marked with red bars. (**A**) **Overview of common vaccines.** Vaccine names are colored to distinguish between those that form the routine, baseline of care (in blue) in PLWH—covering infections that pose a high and constant threat—and those recommended under specific clinical or epidemiological circumstances (in purple). The latter requires an individualized assessment based on factors like immunological status, risk of exposure, age, and comorbidities. (**B**) **Common travel vaccines.** Abbreviations: MMR, mumps, measles, and Rubella; PLWH, people living with HIV infection; RSV, respiratory syncytial virus; Tdap, tetanus, diphtheria, and acellular pertussis; Tp, typhoid.

The advances of HIV therapies and the associated increase in immunocompetence of PLWH are reflected in vaccine recommendations from several organizations and countries that are continuously updated. The most consulted ones are from the World Health Organization (WHO), the U.S. National Institutes of Health (NIH), the Centers for Disease Control and Prevention (CDC), and the European Centre for Disease Prevention and Control (ECDC) [46,78,79,80,81]. Besides these recommendations, vaccination plans are often personalized [75,78,79,82]. This includes regularly testing of antibody levels and giving booster vaccine doses, especially to individuals with low CD4+ T cell counts. An overview of common vaccines and their categories, as well as specific vaccine recommendations for PLWH are given in Figure 2A. We describe the vaccines that are **safe to use**, the ones that are **routine, baseline of care**, vaccines that are **recommended under special circumstances**, and **travel vaccines**.

### 4.1. Vaccines Safe for Use in PLWH

The majority of the vaccines belong to **non-live vaccines.** They contain antigens that cannot replicate and are considered safe and effective for PLWH [83,84,85,86,87,88]. The other group of vaccines is **live-attenuated vaccines** (LAVs). They contain weakened pathogens capable of replication and thus pose safety concerns for immunocompromised individuals. Due to the risk of uncontrolled replication and vaccine-related disease, their administration to PLWH is generally contraindicated for individuals with (i) CD4^+^ T cell counts below 200 cells/µL of blood or (ii) untreated HIV infection [81,88,89,90,91]. However, for patients with higher CD4+ T cell counts (>200–500 cells/µL) and stable viral suppression on HAART, selected live vaccines may be administered after careful risk–benefit assessment and under specialist supervision [89,90]. These vaccines include Zostavax^®^, Varivax^®^, YF-VAX^®^, Stamarli^®^, Vivotif^®^, Vaxchora^®^, ImoJev^®,^ and the BCG vaccine [81,89,90,91,92,93] (Figure 2A). Through these selection criteria, protection against some preventable infections can be provided to PLWH without putting those at risk who are significantly immunosuppressed. Importantly, some LAVs remain contraindicated for all PLWH, regardless of their immune status. These vaccines are (i) the combined measles–mumps–rubella (MMR) + varicella vaccine (ProQuad^®^, Priorix-Tetra^®^), (ii) the intranasal live-attenuated influenza vaccine (FluMist^®^), (iii) the replicating smallpox and monkeypox vaccine ACAM2000^®^, and (iv) the oral poliovirus vaccines (OPVs) (i.e., Orimune^®^, TOPV^®^ (Trivalent Oral Polio Vaccine), Sabin OPV^®^, Polio Sabin™, nOPV2^®^, etc.) [75,81,90,94,95,96,97] (Figure 2A). The reason to avoid these specific vaccines is the option to use safer, non-live vaccine alternatives. Recommended are separate MMR and varicella vaccines, inactivated or recombinant influenza vaccines (IIVs), the non-replicating smallpox and monkeypox JYNNEOS^®^, and the inactivated poliovirus vaccines (i.e., IPOL^®^, IMOVAX^®^ Polio^®^, Poliovax^®^, Sabin IPV^®^, Poliomyelitis Vaccine^®^, Poliovac^®^, etc.) [75,81,90,94,95,96,97].

Of the **live-attenuated vaccines**, the JYNNEOS vaccine against smallpox and monkeypox is a notable exception. It consists of a modified Vaccinia Ankara vector that is incapable of replicating in host cells. While being highly immunogenic, there is no risk of uncontrolled viral spread or reversion to virulence. Therefore, it is very valuable and used to vaccinate PLWH across varying degrees of immune competence [98,99].

### 4.2. Routine, Baseline of Care Vaccines

Since PLWH may vary in their immunocompetence and are at higher risks for severe illnesses caused by diverse respiratory, bloodborne, and sexually transmitted infections, **specific vaccines** are recommended for them (Figure 2A, vaccines in blue). These include annual influenza vaccination [75,78,79,80,81,82,100], SARS-CoV-2 vaccination (with booster doses as indicated) [75,78,79,80,81,82,100], RSV vaccination for adults—especially those aged 60 and older [75,78,79,80,81,82,100], zoster [75,78,79,80,82,100], and pneumococcal vaccination [75,78,79,80,82,100]. Concerning pneumococcal vaccines, current guidelines recommend that PLWH receive a conjugate vaccine—PCV15, PCV20, or PCV21. For individuals vaccinated with PCV15, the vaccination should be followed by an additional dose of the pneumococcal polysaccharide vaccine PPSV23 to broaden serotype coverage. The PPSV23 dose is given at least eight weeks after the PCV15 injection to ensure an optimal immune response and expanded protection [78,79,80,81,82,100]. Moreover, the zoster vaccine, which prevents shingles caused by reactivation of VZV, is recommended for all PLWH aged 19 years and older, regardless of their CD4+ T cell counts [75,78,79,80,82,100]. The quadrivalent meningococcal conjugate vaccine (MenACWY) is also routinely recommended [75,79,82]. The standard schedule often involves a two-dose primary series followed by a booster dose every five years [78,79,80,81,82,100]. Vaccination against Hepatitis A (HAV) and B viruses (HBV), and Human Papillomaviruses (HPV) is recommended. Because PLWH also often have a reduced HBV vaccine response, it is recommended to either use the two-dose HepB-CpG vaccine or higher-dose/extended-dose regimens of traditional vaccines [75,78,79,81,82,100]. To prevent HPV-associated cancers and genital warts, HPV vaccination is strongly advised for PLWH, especially for those aged 18 to 26. Vaccination may be considered up to the age of 45 [78,79,80,82]. Tdap/Td vaccines are important for PLWH to prevent serious disease caused by tetanus, diphtheria, and pertussis. PLWH should receive booster doses of Tdap or Td according to current guidelines [78,79,80,81,82].

### 4.3. Vaccines Recommended Under Special Circumstances

Some vaccines may be indicated **under specific clinical circumstances** (Figure 2A, vaccines in purple) [88,89,101,102]. According to current international guidelines, the *Haemophilus influenzae* type b (Hib) vaccine is no longer routinely recommended for adults unless specific predisposing conditions such as asplenia, sickle-cell disease, or a history of hematopoietic stem cell transplantation are present [100]. However, some protocols advocate Hib vaccination in adults living with HIV who have CD4+ T cell counts above 200 cells/µL as part of broader bacterial infection prevention strategies, even in the absence of additional risk factors [103]. This variability reflects differences among regional guidelines. For instance, European recommendations typically endorse Hib vaccination for children living with HIV (CLHIV), regardless of comorbidities. The reason for this is their heightened vulnerability to invasive bacterial infections [102,104]. MMR vaccination in PLWH is recommended for individuals born in 1957 or after who are not pregnant and lack serological or documented evidence of immunity [75,79,80,82,105]. Varicella vaccination should be administered only to HIV-infected individuals without a history of natural VZV infection, who have not previously been vaccinated, or who received only a single vaccine dose [75,78,79,82]. Meningococcal B vaccination is not routinely indicated for all PLWH but may be considered in those with an increased risk for meningococcal disease, particularly during outbreaks, in regions with high disease prevalence, or in individuals with occupational or behavioral risk factors, for instance, microbiologists, men who have sex with men, and people living in communal settings [79,100]. HIV-infected individuals with an increased risk of invasive meningococcal disease include individuals with functional or anatomic asplenia, including those with sickle-cell disease, as well as those with persistent complement component deficiencies or receiving complement inhibitor therapy (e.g., eculizumab or ravulizumab) [79,100].

BCG vaccination is recommended for neonates who are HIV-exposed but uninfected (HEU), especially in regions with a high burden of tuberculosis (TB). This vaccine provides important protection against severe TB forms in early childhood [106]. However, BCG is contraindicated in infants and children with confirmed HIV infection, regardless of symptoms or immune status. The reason is the significantly increased risk of disseminated BCG disease, a serious complication of the vaccine [106,107,108]. In case virological testing and coverage of HAART are available, it is advised to delay BCG vaccination until HIV infection status is determined [107,109,110]. However, in countries with a high TB burden, for HIV-infected children who are clinically stable and have immune reconstitution on HAART (CD4+T-cell count ≥ 200 cells/μL), BCG vaccination may be considered following specialist assessment [110]. In adults, BCG vaccination is generally not recommended except in specific high-risk situations or exposures. It should always be performed under medical supervision [106]. Finally, vaccination against sexually transmitted infections includes immunization against monkeypox (mpox). It is recommended only for individuals at increased risk of exposure or transmission, such as those with close contact to confirmed cases or within a group of high mpox infection rates, like men who have sex with men [75,78,79,80,82,100]. Targeted vaccination in these populations serves as an important preventive measure to reduce the risk of outbreaks among vulnerable groups.

### 4.4. Travel Vaccines

Important **travel vaccines** for PLWH include those mentioned in Figure 2B [89,90,111,112,113,114]. They should be selected based on destination-specific risks and individual immune status [81,90,111,113,115,116]. PLWH with CD4+ T cell counts below 200 cells/µL or a history of AIDS-defining illnesses are generally advised to delay travel and vaccination until their immunocompetence is restored through effective HAART. Otherwise, the vaccine response may be suboptimal, and the risk of acquiring an infection while traveling is increased [90,111,114,117,118].

## 5. Therapeutic HIV Vaccination Towards a Functional Cure

Besides the absence of an efficacious protective HIV vaccine, there were, and are, numerous trials to use HIV immunogen formulations as an immunotherapy in PLWH. The idea behind these attempts is to improve host immune control over HIV by restoring exhausted immune functions and generating novel ones so that virus replication is kept below pathogenic levels, as, for example, in HIV Elite Controllers (EC) [119,120]. Conceptually, such a functional cure strategy has its theoretical basis in systems biology that predicts multi-stability in biological positive-feedback systems to which virus infections belong [121]. The existence of an apathogenic low-viral-load state has been shown in the lymphocytic choriomeningitis virus (LCMV) model in mice [122]. To achieve it from the high virus load state of chronic infection, the net virus growth rate must be reduced fivefold [122]. This may be achieved through the multiplicative cooperativity of cytotoxic T cells and neutralizing antibodies [123], the latter appearing late during the chronic LCMV infection course [124]. Unfortunately, the analysis of multi-stability in chronic HIV infection is still in its infancy [125], and the means to achieve a stable low virus load state and to maintain it under physiological perturbations are still unclear.

**Figure 3:**
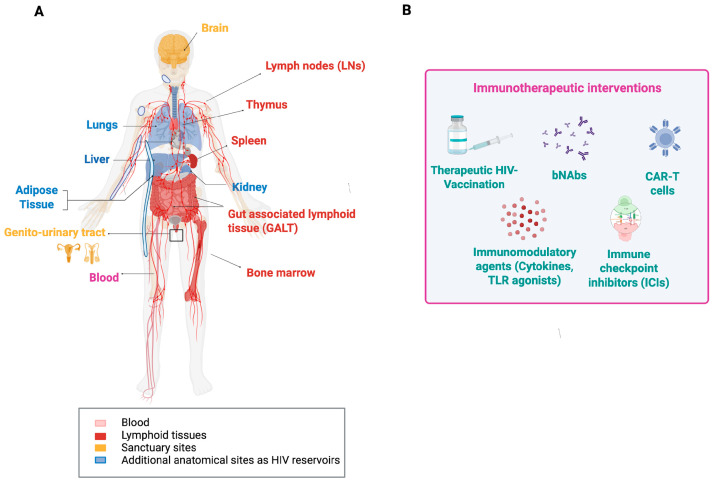
Anatomic locations of HIV and HIV reservoirs (**A**) and immunotherapeutic approaches against HIV (**B**)**.** The main sites for HIV replication are lymphoid tissues. However, for functional cure strategies of HIV infection, other sites and organs in which HIV was detected also need to be considered. The common types of immunological interventions for better controlling HIV infections are depicted in (**B**). Abbreviations: bNAbs, broadly neutralizing antibodies; CAR-T, chimeric antigen receptor T cells; TLR, Toll-like receptor.

A functional HIV cure would reduce the lifelong antiviral therapy dependence and its associated toxicities [126]. Numerous immunotherapeutic trials, both in human and non-human models, have therefore been performed. They include immunotherapeutic agents, therapeutic vaccines, broadly neutralizing antibodies (bNAbs), immune checkpoint inhibitors (ICIs), immunomodulators, and chimeric antigen receptor (CAR) T cells. These agents were used individually or in combinations. To demonstrate effectiveness, immunological and virological endpoints were analyzed, including (i) partial or sustained improvements in T-cell and antibody function, (ii) delayed or reduced viral rebound during antiviral treatment interruptions (ATI), and (iii) a decrease in viral reservoir size. Some of the most recent approaches are summarized below (Figure 3).

Several **therapeutic vaccines** were designed to elicit HIV-specific T-cell responses and enhance virus-infected cell elimination. For instance, the HIVACAT T-cell immunogen (HTI), tested in Phase I/IIa trials (AELIX-002/003) alone and combined with the TLR7 agonist vesatolimod, generated strong HTI-specific T-cell responses [127,128]. While viral rebound occurred in all participants during ATI, approximately one-third of those receiving the combination maintained better viral control and experienced a prolonged time off antiretroviral therapy, which correlated directly with HTI-specific T-cell levels [127]. Other candidates, such as Vacc-4x and tHIVconsvX, have also shown some promise, especially when paired with immunomodulators [129,130]. Nonetheless, vaccine-induced levels of HIV-specific T-cell responses from all these trials have proven insufficient to maintain long-term virus control after ATI. This may not come as a surprise since the predicted threshold level of HIV-specific cytotoxic T cells required to detect productively infected cells within lymphatic tissue before they release virus particles is around 5% of all CD8+ T cells [131]. To then prevent virus release, the CTLs also need to be fully functional and kill the infected cell before that happens. This estimate of 5% is derived from a mathematical model analyzing in vivo imaging data of lymphocyte migration within lymphatic tissue. That model did not take HIV variation, T-cell receptor clonality, or CD8 T-cell exhaustion into account. Consequently, the actual threshold level for HIV control might be significantly higher. Together with an observed Gag-specific CTL frequency of up to 1% in lymph nodes of HIV-1 carriers [132], the required CTL threshold level for HIV control might be extremely difficult to achieve with T-cell-based vaccines alone.

HIV-specific **broadly neutralizing antibodies (bNAbs)** are a class of antibodies that appear in some PLWH. They target conserved regions of the HIV envelope and can neutralize diverse viral strains [133]. Several such antibodies have been manufactured and tested in combination or alone in clinical trials for their capacity to suppress viremia and delay virus rebound after HAART interruption [134,135,136,137]. Combined bNAbs, such as the triple antibody regimen PGT121 plus PGDM1400 and VRC07-523LS, maintained viral suppression longer than single antibodies, paralleling the combinatorial approach seen with therapeutic vaccines [138]. Interestingly, bNAbs engage immune effector functions beyond virus neutralization, for example, antibody-dependent cellular cytotoxicity (ADCC) and phagocytosis to clear infected cells and modulate immune activation [139,140]. They are also associated with increased virus-specific T-cell immunity [137], which is crucial for long-term control. Besides this, bNAbs are confronted by several challenges, such as virus immune escape due to within-host HIV diversity, production costs, and dosing logistics [133,138,141,142]. Nonetheless, leading candidates are advancing through engineered half-life extensions and combination with immunomodulators such as TLR agonists and IL-15 superagonists [142,143,144,145,146]. While bNAbs still await regulatory approval, they are, alongside therapeutic vaccines, considered an essential component of HIV immunotherapy strategies [133,142,147,148].

Immune cell exhaustion is a fundamental component of chronic viral infections and one of the barriers to a functional HIV cure. Therefore, immunotherapeutic strategies, including **immune checkpoint inhibitors** (ICI) and **immunomodulators**, such as TLR agonists and cytokines, are also being intensively investigated [149,150,151,152]. For the treatment of malignancies in PLWH, ICIs like pembrolizumab (anti-PD-1) and ipilimumab (anti-CTLA-4) are already approved, but their use in HIV cure strategies remains experimental [149]. Theoretical considerations have suggested that the results of ICI treatment against HIV would depend on the phenotype of the infection and the combined antiviral activity of adaptive immunity other than T cells [153]. Early clinical work indicated that ICIs are generally safe in PLWH and may modestly reduce immune exhaustion or impact the viral reservoir. However, results are inconsistent and require further validation [149,154]. **TLR7/8 agonists** (e.g., vesatolimod) are under evaluation for their ability to reverse HIV latency and stimulate immune responses. They are well tolerated and induce viral transcription and immune activation. There is also some evidence of a delayed viral rebound after treatment interruption, although a consistent reservoir reduction has not yet been demonstrated [155,156,157]. Similarly, cytokines such as the **IL-15 superagonist** N-803 are investigated for latency reversal and boosting cytotoxic lymphocyte function. However, they remain investigational and are still unlicensed for HIV treatment [158,159].

Finally, an exciting new strategy towards a functional cure for HIV is based on **CAR T cells**. The idea behind it is a reprogramming of patients’ own T cells with chimeric antigen receptors (CARs) that recognize key parts of HIV and then attack and destroy HIV-infected cells [160,161]. CAR T cell therapy was shown to be highly effective in some cancer treatments and may enable targeting HIV reservoir cells in PLWH [160,162]. A promising advance is the “duoCAR” design. It uses two receptors that target different conserved HIV envelope sites to enhance immune breadth and minimize viral immune escape. Preclinical humanized mouse models demonstrate > 97% viral suppression and delayed rebound post-HAART interruption [163,164]. To further improve CAR T cell effectiveness and increase their lifespan, elements from bNAbs are integrated, and the HIV-co-receptor CCR5 is eliminated to render them infection-resistant [165,166,167]. While the high costs are a current barrier [168], global research efforts are intensifying to make this powerful therapy more effective, durable, and accessible. Combined with other immunotherapies, CAR T cells could play a key role in the next generation of HIV cure strategies [160,165,166].

Taken together, the most compelling option for a functional cure of an HIV infection involves synergistic combinations of multiple immunotherapies that utilize the strengths of therapeutic vaccines, bNAbs, immune activators, and CAR T cells. However, safety concerns such as immune-related side effects and inflammation require careful patient monitoring [149,169,170]. Balancing efficacy with minimal toxicity will be crucial for bringing these therapies into clinical use.

## 6. Conclusions

Vaccination of PLWH presents unique challenges, largely driven by varying levels of immunosuppression and dependence on antiretroviral therapy. While effective HAART has significantly improved vaccine outcomes, individuals with lower CD4+ T cell counts require tailored vaccination strategies and immune-response monitoring. Current recommendations favor non-live vaccines for PLWH, with live-attenuated vaccines being reserved for those with higher CD4+ T cell counts. Ongoing advances in immunotherapies, such as therapeutic vaccines, broadly neutralizing antibodies, immune checkpoint inhibitors, immunomodulators, and CAR T cell technology, hold promise to strengthen HIV-specific immunity and may pave the way towards a functional cure. Nonetheless, further research is required to define broadly applicable synergistic combinations of immunotherapeutic modalities and to maintain long-term immune-mediated viral control.

## Data Availability

Data sharing is not applicable.
